# Comprehensive behavioral phenotyping of a new Semaphorin 3 F mutant mouse

**DOI:** 10.1186/s13041-016-0196-4

**Published:** 2016-02-09

**Authors:** Ikuo Matsuda, Hirotaka Shoji, Nobuyuki Yamasaki, Tsuyoshi Miyakawa, Atsu Aiba

**Affiliations:** Division of Cell Biology, Department of Molecular and Cellular Biology, Kobe University Graduate School of Medicine, Kobe, Hyogo 650-0017 Japan; Department of Surgical Pathology, Hyogo College of Medicine, Nishinomiya, Hyogo 663-8501 Japan; Division of Systems Medical Science, Institute for Comprehensive Medical Science, Fujita Health University, Toyoake, Aichi 470-1192 Japan; Kyoto Prefectural Rakunan Hospital, 2 Hirookadani, Gokasho, Uji, Kyoto 611-0011 Japan; Genetic Engineering and Functional Genomics Group, Horizontal Medical Research Organization (HMRO), Kyoto University Graduate School of Medicine, Sakyo-ku, Kyoto 606-8501 Japan; Section of Behavior Patterns, Center for Genetic Analysis of Behavior, National Institute for Physiological Sciences, Okazaki, Aichi 444-8585 Japan; Laboratory of Animal Resources, Center for Disease Biology and Integrative Medicine, Faculty of Medicine, The University of Tokyo, Bunkyo-ku, Tokyo, 113-0033 Japan

**Keywords:** Semaphorin 3 F, Knockout mice, Anxiety, Fear, A behavioral test battery

## Abstract

**Background:**

Semaphorin 3 F (Sema3F) is a secreted type of the Semaphorin family of axon guidance molecules. Sema3F and its receptor neuropilin-2 (Npn-2) are expressed in a mutually exclusive manner in the embryonic mouse brain regions including olfactory bulb, hippocampus, and cerebral cortex. Sema3F is thought to have physiological functions in the formation of neuronal circuitry and its refinement. However, functional roles of Sema3F in the brain remain to be clarified. Here, we examined behavioral effects of Sema3F deficiency through a comprehensive behavioral test battery in *Sema3F* knockout (KO) male mice to understand the possible functions of *Sema3F* in the brain.

**Results:**

Male *Sema3F* KO and wild-type (WT) control mice were subjected to a battery of behavioral tests, including neurological screen, rotarod, hot plate, prepulse inhibition, light/dark transition, open field, elevated plus maze, social interaction, Porsolt forced swim, tail suspension, Barnes maze, and fear conditioning tests. In the open field test, *Sema3F* KO mice traveled shorter distance and spent less time in the center of the field than WT controls during the early testing period. In the light/dark transition test, *Sema3F* KO mice also exhibited decreased distance traveled, fewer number of transitions, and longer latency to enter the light chamber compared with WT mice. In addition, *Sema3F* KO mice traveled shorter distance than WT mice in the elevated plus maze test, although there were no differences between genotypes in open arm entries and time spent in open arms. Similarly, *Sema3F* KO mice showed decreased distance traveled in the social interaction test. *Sema3F* KO mice displayed reduced immobility in the Porsolt forced swim test whereas there was no difference in immobility between genotypes in the tail suspension test. In the fear conditioning test, *Sema3F* KO mice exhibited increased freezing behavior when exposed to a conditioning context and an altered context in absence of a conditioned stimulus. In the tests for assessing motor function, pain sensitivity, startle response to an acoustic stimulus, sensorimotor gating, or spatial reference memory, there were no significant behavioral differences between *Sema3F* KO and WT mice.

**Conclusions:**

These results suggest that Sema3F deficiency induces decreased locomotor activity and possibly abnormal anxiety-related behaviors and also enhances contextual memory and generalized fear in mice. Thus, our findings suggest that Sema3F plays important roles in the development of neuronal circuitry underlying the regulation of some aspects of anxiety and fear responses.

## Background

Semaphorins are a family of secreted or membrane-bound molecules containing an around 500-amino acid extracellular domain termed semaphorin domain. The semaphorins are subdivided into eight classes, of which classes 3-7 are found in vertabrates [1]. The class 3 subfamily of semaphorins, originally identified as axon guidance molecules, is expressed throughout the brain and has crucial roles in synapse formation and plasticity [2]. Despite the advances in the understanding of some semaphorins and their downstream signaling pathways that are implicated in neural circuit development [2], there is relatively limited information about contributions of those molecules to brain function and behavior [3–5].

Semaphorin 3 F (Sema3F), one of the class 3 semaphorins, is a secreted protein that binds to a transmembrane receptor protein Neuropilin-2 (Npn-2) [6, 7]. The Sema3F/Npn-2 interaction evokes intracellular signals for axon guidance via the Plexin family protein(s) [2]. The mRNAs for Sema3F and its receptor neuropilin-2 (Npn-2) are expressed in a mutually exclusive manner in the embryonic mouse brain regions including olfactory bulb, hippocampus, and cerebral cortex [8]. To examine physiological roles of Sema3F during developing brain, we and others investigated physiological properties in postnatal brain of *Sema3F* knockout (KO) mice or *Npn-2* KO mice. It was demonstrated that *Sema3F* KO and *Npn-2* KO mice showed various neuroanatomical defects in neuronal wiring and/or its synaptic transmission in brain regions such as olfactory bulb, hippocampus, cortex, and habenula [8–14]. It is reported that *Sema3F* and *Npn-2* KO mice exhibit increased spine number and size in the granule cells in the dentate gyrus (DG) of the hippocampus and cortical layer V pyramidal neurons and also show increased frequency of miniature excitatory postsynaptic current, indicating that Sema3F/Npn-2 signaling is a negative regulator of spine development and synaptic structure [12]. Taken together, Sema3F/Npn-2 signaling has essential roles in neuronal circuitry formation during brain development. However, it remains to be examined whether the neuroanatomical aberrations induced by abnormal Sema3F expression lead to changes in behavior.

In the present study, to reveal the functional and behavioral roles of Sema3F in brain, we examined various behaviors of *Sema3F* KO mice through a battery of behavioral tests, including neurological screen, light/dark transition, open field, elevated plus maze, hot plate, social interaction, rotarod, prepulse inhibition, Porsolt forced swim, fear conditioning, tail suspension, and Barnes maze tests. Our results of behavioral analysis indicate that *Sema3F* KO mice show abnormal anxiety-related behaviors in the open field and light/dark transition tests and enhanced freezing in the conditioning context and altered context of the fear conditioning test, suggesting that Sema3F is involved in regulation of innate and learned fear.

## Results

### *Sema3F* KO mice exhibit normal sensorimotor function

**Fig. 1:**
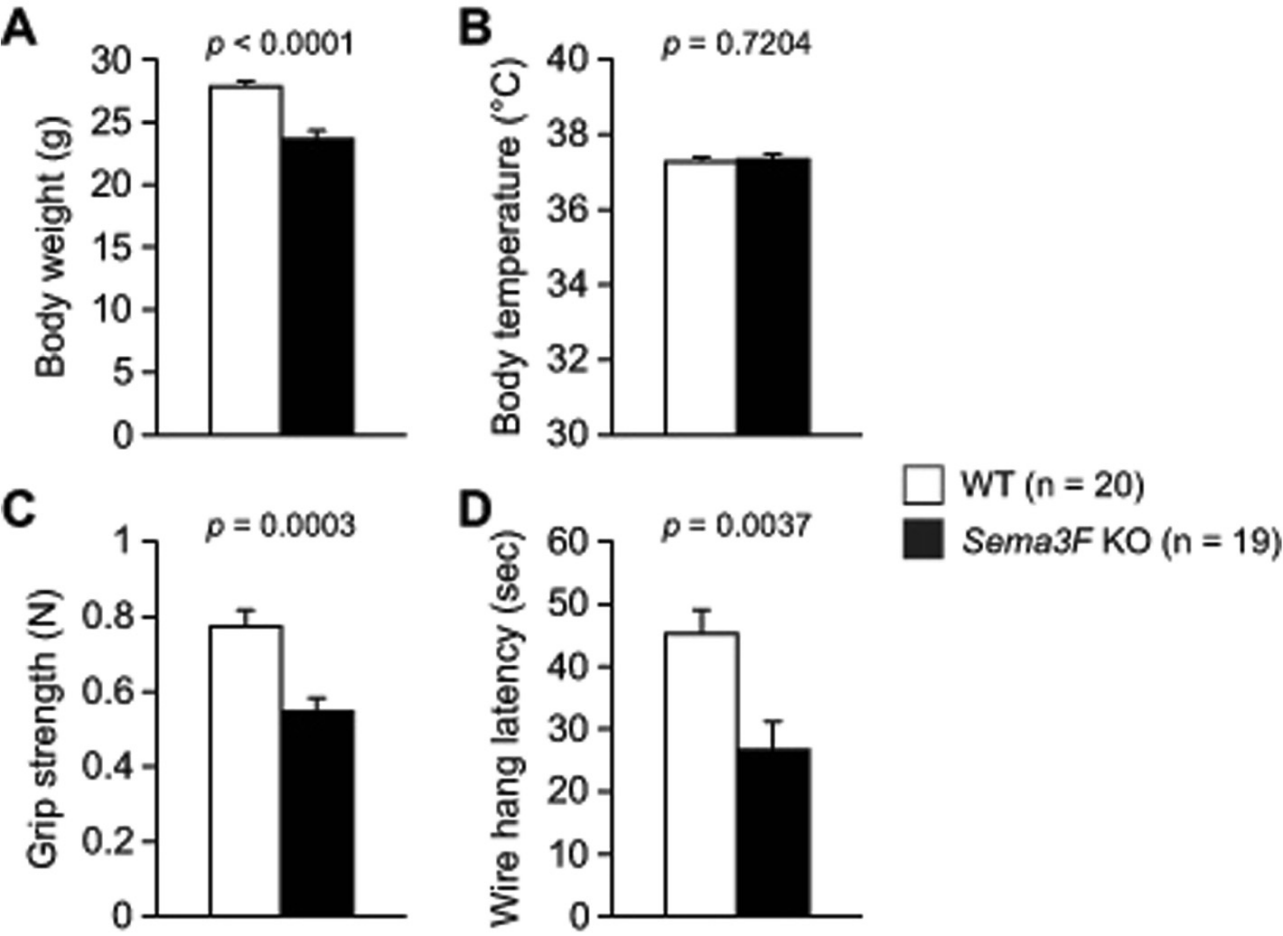
General health and neurological screen. **a** body weight, (**b**) body temperature, (**c**) grip strength, and (**d**) wire hang latency. Data indicate means ± SEM (n = 19 for *Sema3F* KO mice; n = 20 for WT controls)

We compared general health and neurological characteristics of *Sema3F* KO mice with those of WT control mice. The appearance of fur and whiskers and the neurological reflexes were normal in *Sema3F* KO mice. *Sema3F* KO mice exhibited significantly lighter body weight (Fig. 1a, *t*_37_ = 4.996, *p* < 0.0001), weaker grip strength (Fig. 1c, *t*_37_ = 3.955, *p* = 0.0003), and shorter wire-hang latency (Fig. 1d, *t*_37_ = 3.103, *p* = 0.0037) than WT control mice. There was no significant difference between the genotypes in body temperature (Fig. 1b, *t*_37_ = 0.361, *p* = 0.7204). These results show that Sema3F is required for physical and neuromuscular development.

**Fig. 2:**
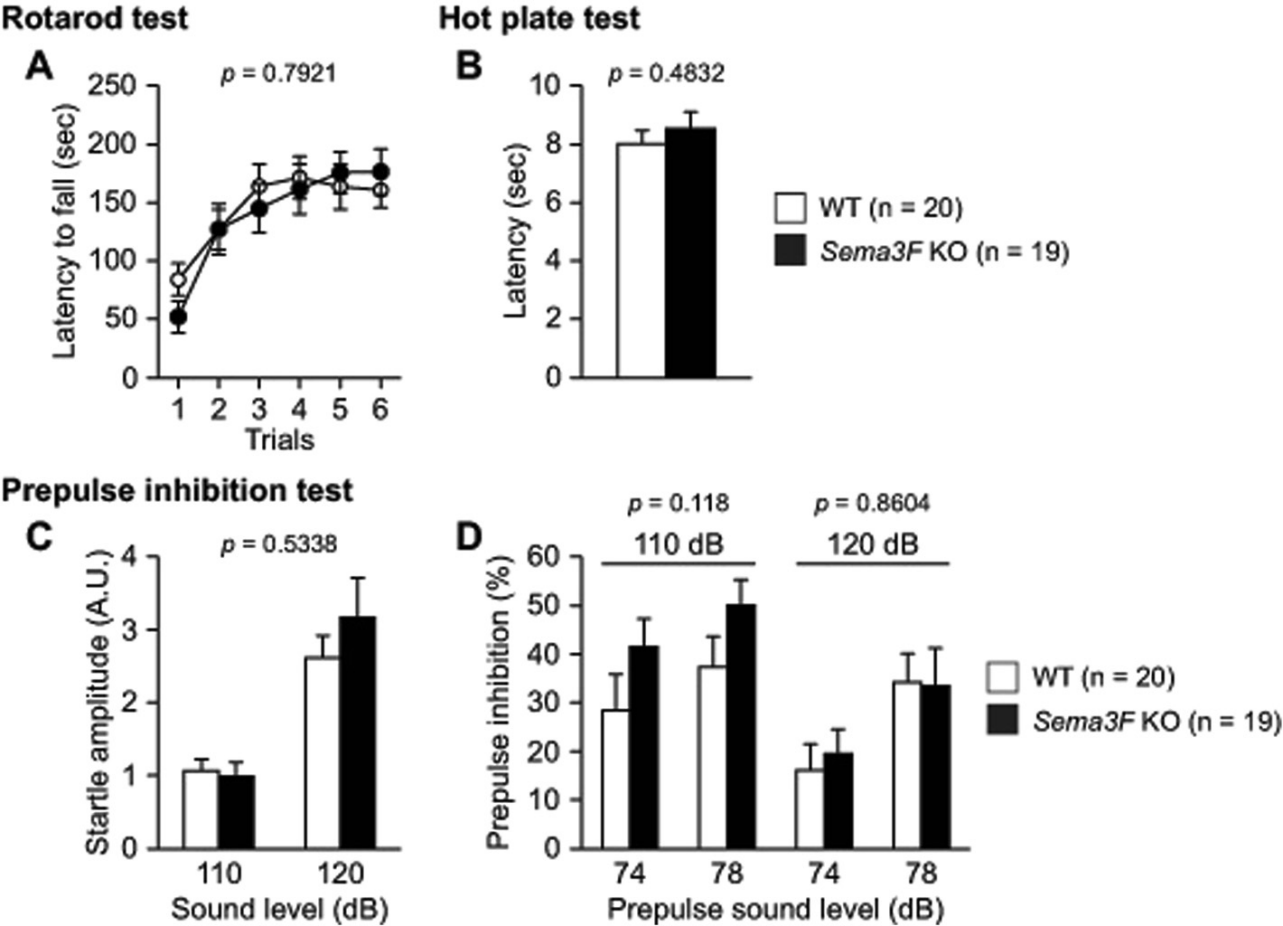
Normal motor coordination, pain sensitivity and sensorimotor function in *Sema3F* KO mice. **a** The latency to fall from an accelerating rotarod was measured by three trials per day for two consecutive days in the rotarod test. There was no significant difference in the latency to fall between *Sema3F* KO mice and WT controls. **b** The hot plate test was used to evaluate sensitivity to a painful stimulus. Mice were placed on a hot plate and latency to the first hindpaw response was recorded. There was no significant difference in the latency between the genotypes. **c**, **d** The startle response/prepulse inhibition tests were performed to examine startle responses to loud stimuli (110 or 120 dB) and inhibition of the startle response by prepulse stimulus (74 or 78 dB). **c** The startle response and (**d**) the prepulse inhibition were not significantly different between *Sema3F* KO mice and WT controls. Data indicate means ± SEM (n = 19 for *Sema3F* KO mice; n = 20 for WT controls)

There were no significant differences between *Sema3F* KO and WT control mice in rotarod performance (Fig. 2a, *F*_1, 37_ = 0.07, *p* = 0.7921), hot plate latency (Fig. 2b, *t*_37_ = 0.708, *p* = 0.4832), startle responses to 110 and 120 dB stimuli (Fig. 2c, *F*_1, 37_ = 0.394, *p* = 0.5338), or prepulse inhibition of the startle response (Fig. 2d, for 110 dB startle, *F*_1, 37_ = 2.561, *p* = 0.118; for 120 dB startle, *F*_1, 37_ = 0.031, *p* = 0.8604). These observations indicate that *Sema3F* KO mice have no deficits in sensory or motor functions.

### *Sema3F* KO mice exhibit increased anxiety-related behavior

**Fig. 3:**
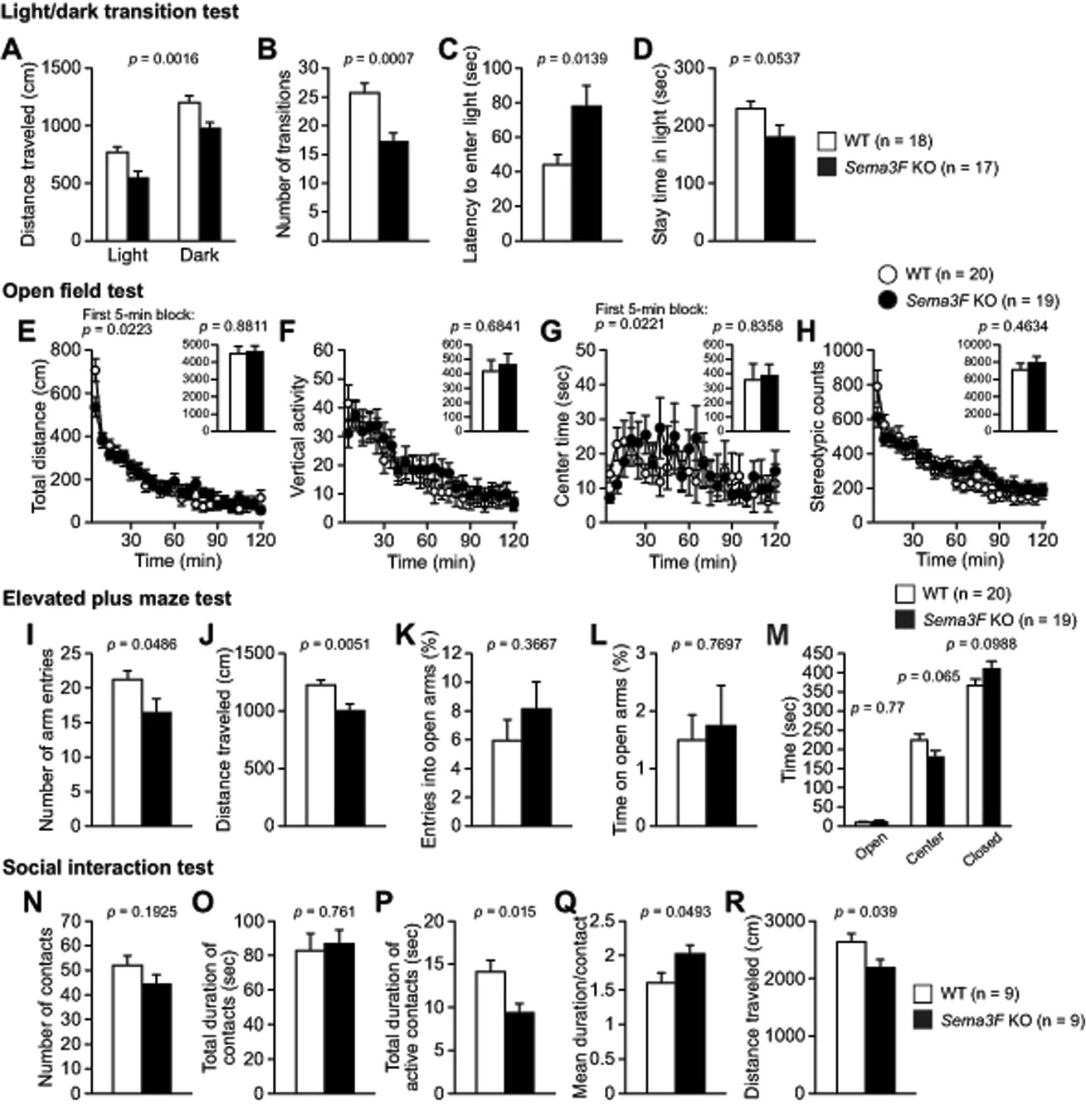
Decreased locomotor activity and increased anxiety-related behavior in *Sema3F* KO mice. **a**-**d** Light/dark transition test. **a** Distance traveled, (**b**) number of transitions between the light and dark chambers, (**c**) latency to enter the light chamber, and (**d**) stay time in the light chamber (n = 17 for *Sema3F* KO mice; n = 18 for WT controls). **e**-**h** Open field test. **e** Distance traveled, (**f**) vertical activity, (**g**) time spent in center area, and (**h**) stereotypic behavior counts (n = 19 for *Sema3F* KO mice; n = 20 for WT controls). **i**-**m** Elevated plus maze test. **i** Number of total entries into arms, (**j**) distance traveled, (**k**) percentage of entries into open arms, (**l**) percentage of time spent in open arms, and (**m**) time spent in open arms, center area, and closed arms (n = 19 for *Sema3F* KO mice; n = 20 for WT controls). **n**-**r** Social interaction test in a novel environment. **n** Number of contacts, (**o**) total duration of contacts, (**p**) total duration of active contacts, (**q**) mean duration per contact, and (**r**) distance traveled (n = 9 for each genotype)

*Sema3F* KO and WT control mice were compared in behavioral tests for assessing anxiety-related behavior, including light/dark transition, open field, elevated plus maze, and social interaction tests. In the light/dark transition test, *Sema3F* KO mice showed decreased distance traveled, fewer number of transitions between the light and dark chambers, and shorter latency to enter the light chamber compared with WT mice (Fig. 3a, for distance traveled in the light and dark chambers, Genotype effect, *F*_1,33_ = 11.871, *p* = 0.0016; Genotype × Chamber interaction, *F*_1,33_ = 0.00005, *p* = 0.9944; Fig. 3b, for number of transitions, *t*_33_ = 3.763, *p* = 0.0007; Fig. 3c, for latency to enter the light chamber, *t*_33_ = 2.597, *p* = 0.0139). Moreover, *Sema3F* KO mice showed a trend to stay shorter time in the light chamber than WT controls (Fig. 3d, *t*_33_ = 2.001, *p* = 0.0537). These results indicate that Sema3F deficiency induces increased anxiety-related behavior.

In the open field test, there were no significant genotype differences in the total distance traveled, vertical activity, center time, or stereotypic counts during 120-min test period (Fig. 3e, for total distance traveled, Genotype effect, *F*_1,37_ = 0.023, *p* = 0.8811; Genotype × Time interaction, *F*_23,851_ = 1.845, *p* = 0.0093; Fig. 3f, for vertical activity, Genotype effect, *F*_1, 37_ = 0.168, *p* = 0.6841; Genotype × Time interaction, *F*_23, 851_ = 0.807, *p* = 0.7254; Fig. 3g, for center time, Genotype effect, *F*_1, 37_ = 0.044, *p* = 0.8358; Genotype × Time interaction, *F*_23, 851_ = 1.321, *p* = 0.143; Fig. 3h, for stereotypic counts, Genotype effect, *F*_1, 37_ = 0.549, *p* = 0.4634; Genotype × Time interaction, *F*_23, 851_ = 1.891, *p* = 0.007). However, in the first 5-min period of the test, during which anxiety-like behavior has been generally assessed [15], *Sema3F* KO mice traveled a shorter distance and stayed less time in the center area compared with WT controls (Fig. 3e, for distance traveled, *p* = 0.0223; Fig. 3g, for center time, *p* = 0.0221).

In the elevated plus maze test, *Sema3F* KO mice showed a significantly smaller number of total entries into arms and traveled a significantly shorter distance than WT control mice (Fig. 3i, for number of total arm entries, *t*_37_ = 2.039, *p* = 0.0486; Fig. 3j, for distance traveled, *t*_37_ = 2.98, *p* = 0.0051). The *Sema3F* KO mice spent longer time in closed arms and less time in center area than WT controls although the difference did not reach a statistical significance (Fig. 3m, for time spent in closed arms, *t*_37_ = 1.693, *p* = 0.0988; for time spent in center area, *t*_37_ = 1.902, *p* = 0.065). These results indicate decreased locomotor activity in *Sema3F* KO mice in novel environment, which appear to be consistent with the findings of the light/dark transition test and open field test. However, there were no significant differences between the genotypes in time spent in open arms (Fig. 3m, *t*_37_ = 0.295, *p* = 0.77), percentage of entries into open arms (Fig. 3k, *t*_37_ = 0.914, *p* = 0.3667), or percentage of time spent in open arms (Fig. 3l, *t*_37_ = 0.295, *p* = 0.7697).

In the social interaction test in a novel environment, total duration of active social contacts and distance traveled were significantly decreased in *Sema3F* KO mice compared with WT control mice (Fig. 3p, for total duration of active contacts, *t*_16_ = 2.726, *p* = 0.015; Fig. 3r, for distance traveled, *t*_16_ = 2.249, *p* = 0.039). The mean duration per contact was increased in *Sema3F* KO mice (Fig. 3q, *t*_16_ = 2.128, *p* = 0.0493). There were no significant differences in total number of contacts (Fig. 3n, *t*_16_ = 1.36, *p* = 0.1925) or total duration of contacts (Fig. 3o, *t*_16_ = 0.309, *p* = 0.761). These results show an altered locomotor activity under this novel environment with a stranger mouse, but failed to show abnormality in social behavior per se in *Sema3F* KO mice.

Together, these results from the different types of tests for assessing anxiety-related behavior indicate that *Sema3F* KO mice exhibit decreased locomotor activity and abnormal anxiety-related behaviors in novel environments.

### *Sema3F* KO mice show hyperactivity in forced swim test

**Fig. 4:**
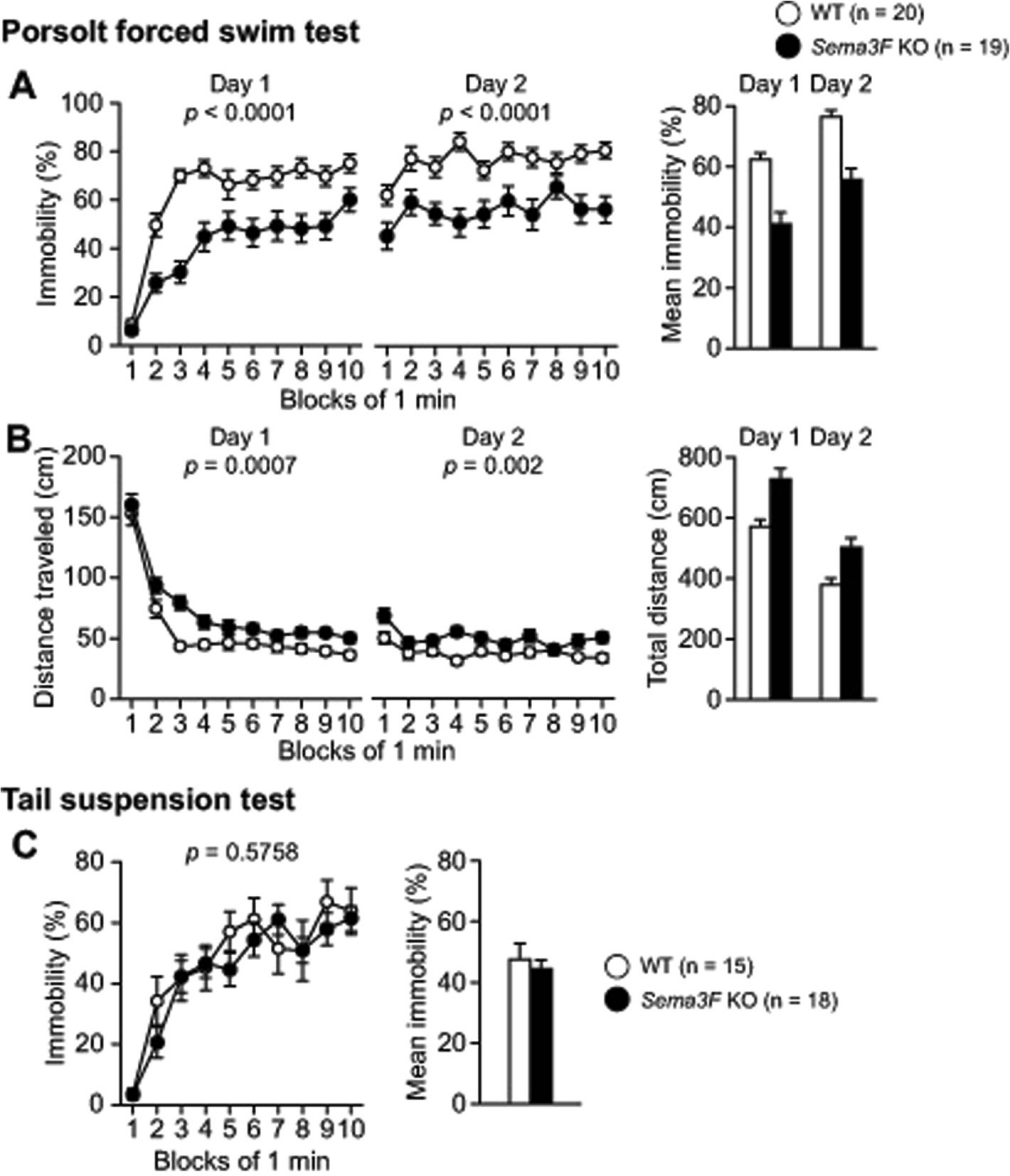
Hyperactivity in forced swim test in *Sema3F* KO mice. **a**, **b** Porsolt forced swim test. Mice were placed in a water-filled cylinder, and immobility time was measured every 1 min for a 10-min period on the first and second days. *Sema3F* KO mice spent significantly less time in immobility (**a**) and traveled significantly longer distances (**b**) than their WT controls. N = 19 for *Sema3F* KO mice; N = 20 for WT controls. **c** Tail suspension test. There was no significant difference in immobility between *Sema3F* KO mice and WT controls. All data indicate means ± SEM (n = 18 for *Sema3F* KO mice; n = 15 for WT controls)

Depression-related behavior was evaluated by the Porsolt forced swim and tail suspension tests. In the Porsolt forced swim test, *Sema3F* KO mice displayed significantly less immobility and traveled significantly longer distances than their WT controls (Fig. 4a, for immobility on Day 1, *F*_1, 37_ = 24.035, *p* < 0.0001; for immobility on Day 2, *F*_1, 37_ = 23.318, *p* < 0.0001; Fig. 4b, for distance traveled on Day 1, *F*_1, 37_ = 13.735, *p* = 0.0007; for distance traveled on Day 2, *F*_1, 37_ = 11.053, *p* = 0.002). In the tail suspension test, there was no significant difference in immobility between *Sema3F* KO and WT control mice (Fig. 4c, *F*_1, 31_ = 0.32, *p* = 0.5758). These findings suggest that Sema3F deficiency may induce hyperactivity in forced swim tests. The discrepancy between forced swim and tail suspension tests indicate that the results may not simply reflect decreased depression-related behavior (see Discussion below).

### *Sema3F* KO mice exhibit increased freezing in the conditioning context and altered context

**Fig. 5:**
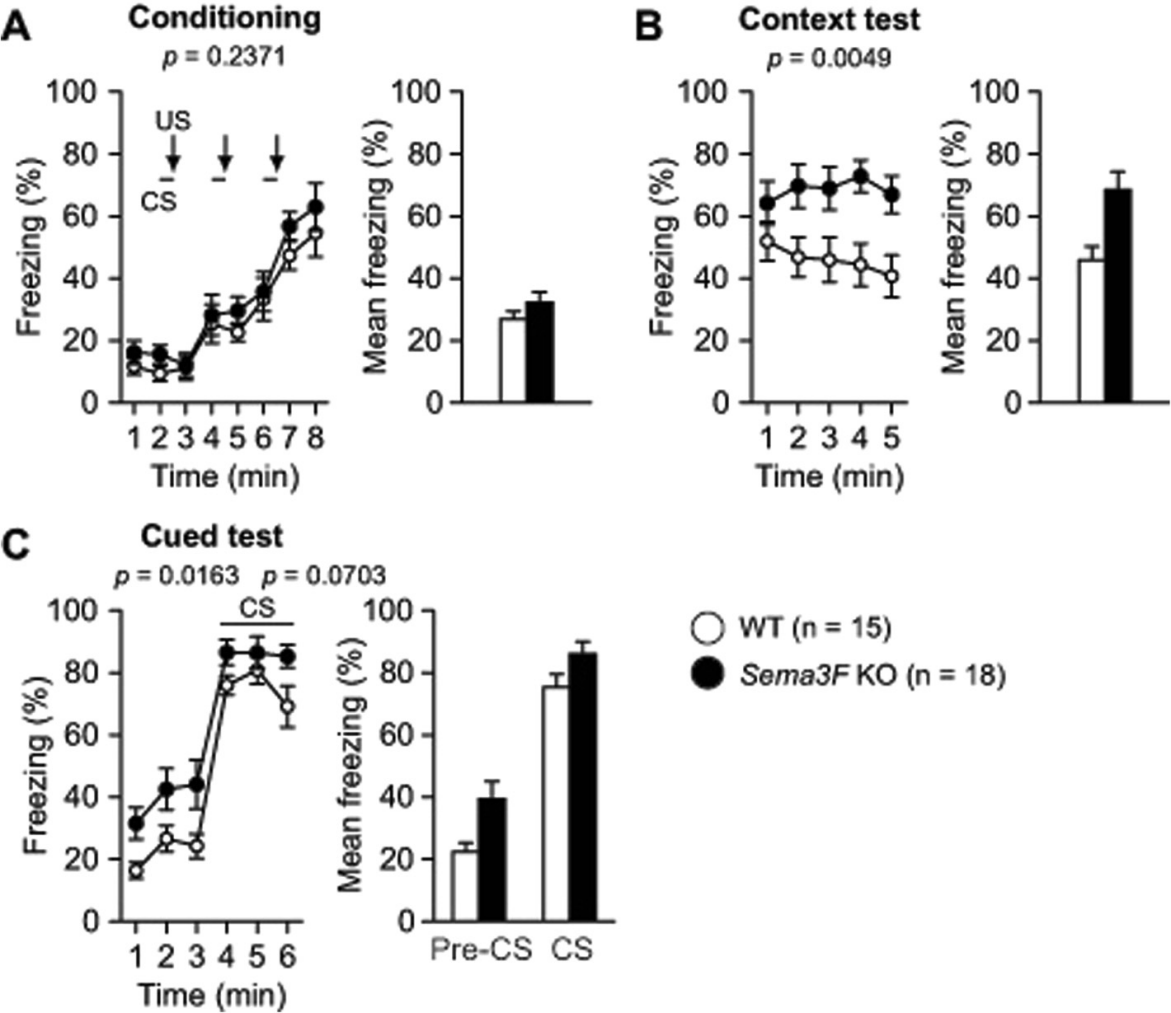
Increased fear memory in *Sema3F* KO mice. Freezing behavior was measured in the contextual and cued fear conditioning test to assess fear memory in *Sema3F* KO mice. **a** There were no significant differences in freezing between *Sema3F* KO mice and WT controls during the conditioning session. **b**, **c** *Sema3F* KO mice exhibited more freezing than WT controls in the re-exposure to the same context approximately 24 hours after the conditioning session and in the altered context with absence or presence of the auditory cue. Data indicate means ± SEM (n = 18 for *Sema3F* KO mice; n = 15 for WT controls)

To assess memory performance in *Sema3F* KO and WT control mice, mice were conditioned with three pairs of auditory cue (conditioned stimulus, CS) and footshock (unconditioned stimulus, US) in the conditioning session. Twenty-four hours after the conditioning, they were tested to assess fear memory by exposure to the conditioning context (context test) followed by the altered context without and with the auditory cue (cued test). There was no significant difference in the percentage of freezing between the genotypes during the conditioning session (Fig. 5a, *F*_1, 31_ = 1.453, *p* = 0.2371). In the context test, *Sema3F* KO mice showed a significantly more freezing behavior than WT controls in the re-exposure to the same context of the conditioning session (Fig. 5b, *F*_1, 31_ = 9.163, *p* = 0.0049). In the cued test with altered context, *Sema3F* KO mice exhibited significantly more freezing than WT controls in the absence of the CS (Fig. 5c, *F*_1, 31_ = 6.447, *p* = 0.0163). During the cued test, *Sema3F* KO mice also showed more freezing than WT controls in presence of the CS although the difference failed to reach a statistical significance (Fig. 5c, *F*_1, 31_ = 3.514, *p* = 0.0703). These results suggest that Sema3F deficiency causes increased generalized fear as well as increased contextual fear memory.

### *Sema3F* KO mice show normal spatial reference memory

**Fig. 6:**
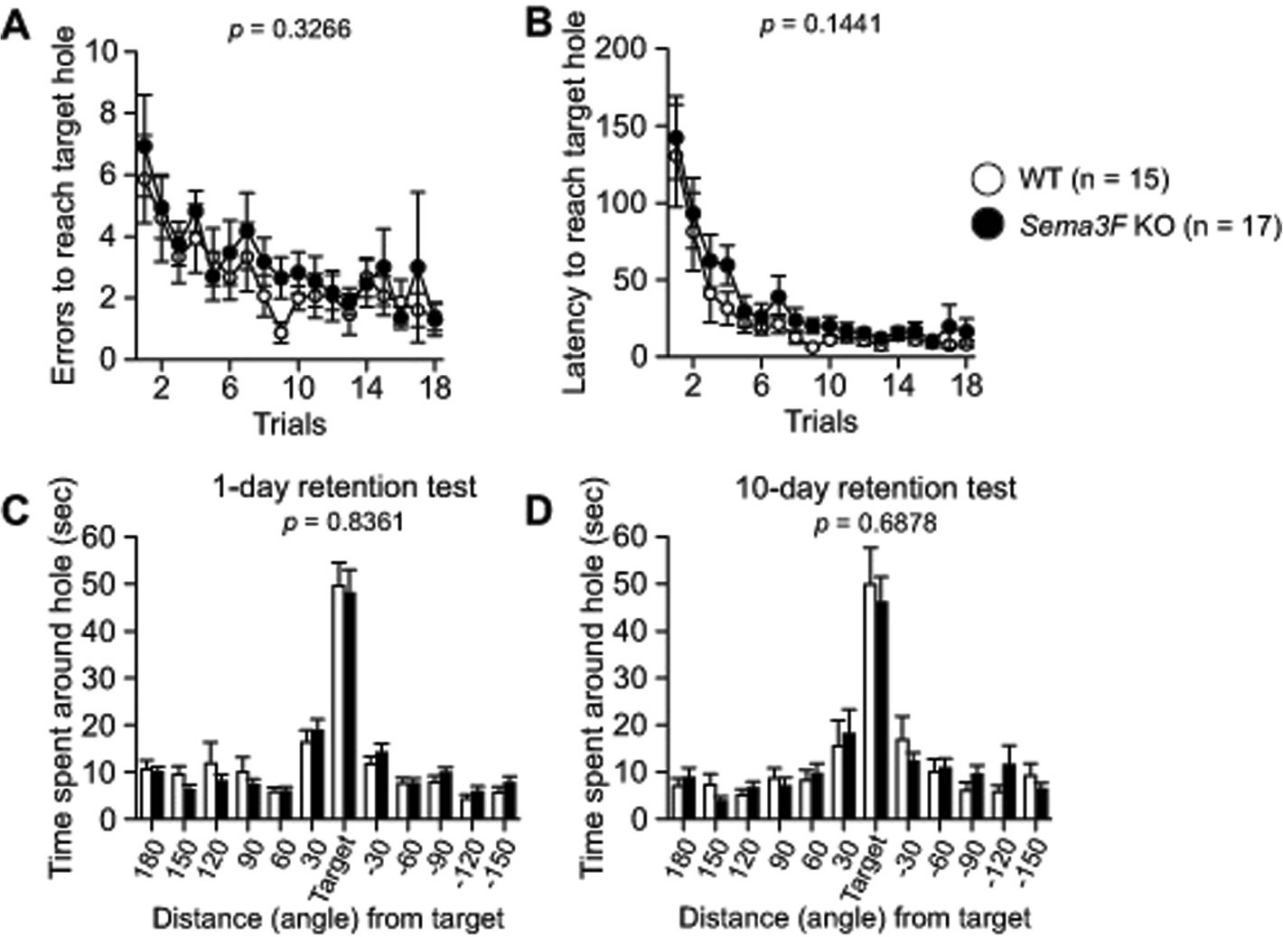
Normal spatial reference memory in *Sema3F* KO mice. Spatial reference memory was tested in the Barnes circular maze. **a**, **b** During the training session, there were no significant differences in the number of errors and latency to reach the correct target hole between *Sema3F* KO mice and WT controls. **c**, **d** In the retention probe tests, 1 day and 10 days after the last training session, *Sema3F* KO mice and WT controls spent similar time in the correct target hole. Data indicate means ± SEM (n = 18 for *Sema3F* KO mice; n = 15 for WT controls)

Spatial reference memory in *Sema3F* KO and WT control mice was tested in the Barnes circular maze. In the training session, there were no significant differences between the genotypes in the number of error to reach the correct target hole or latency to reach the correct target hole (Fig. 6a, *F*_1, 30_ = 0.995, *p* = 0.3266; Fig. 6b, *F*_1, 30_ = 2.249, *p* = 0.1441, respectively), indicating normal spatial learning in *Sema3F* KO mice. One day and 10 days after the last training session, probe tests were performed to evaluate the retention of spatial memory. There were no significant differences between *Sema3F* KO and WT control mice in the time spent around the target hole in either 1- or 10-day retention probe tests (Fig. 6c, for 1 day later, *t*_30_ = 0.209, *p* = 0.8361; for 10 days later, *t*_30_ = 0.406, *p* = 0.6878). These findings indicate that spatial learning and memory in *Sema3F* KO mice are normal.

## Discussion

The present study examined behaviors of *Sema3F* KO male mice using a battery of behavioral tests. To our knowledge, this is the first report on the comprehensive behavioral analysis of mutant mice lacking the class 3 semaphorin. Our physical and behavioral assessments revealed that *Sema3F* KO mice showed no differences in the hot plate latency, rotarod latency, acoustic startle response, and prepulse inhibition of the startle response compared with WT mice, indicating normal sensory and motor functions and sensorimotor gating. Decreased body weight, grip strength, and wire hang latency were observed in *Sema3F* KO mice, indicating that the KO mice exhibited reduced neuromuscular strength. However, *Sema3F* KO and WT mice showed no differences in total distance traveled and vertical activity during the entire period of the open field test in addition to rotarod performance. Thus, it is not likely that the decreased neuromuscular strength affect motor function and general locomotor activity in *Sema3F* KO mice. This conclusion seems to be consistent with the previous study showing that there are no significant differences between *Sema3F* KO and WT control mice in anatomical structures in the somatosensory cortex or cerebellum [8], underlying the control of motor functions. These results seem to be consistent with the findings of a recent study reporting that mice lacking *Npn-2*, a Sema3F receptor, exhibited normal locomotor activity and hot plate latency, compared with their control mice [5]. However, *Npn-2* KO mice showed impaired rotarod performance at the trial 8 and 10, while there were no significant differences in the performances from trial 1 to 7 compared with heterozygous control mice [5]. What causes slightly different results of rotarod test between *Sema3F* KO mice and *Npn-2* KO mice? The Sema3F receptor is considered to be a heteromeric complex containing Npn-2 and Plexin A [16]. Npn-2, when ectopically expressed in COS cells, binds to Sema3B, Sema3C, and Sema3G as well as Sema3F [16]. The observed differences between behavioral phenotypes of *Sema3F* KO mice in this study and that of *Npn-2* KO mice [5] may reflect the loss of binding of Sema3B, Sema3C, and Sema3G to Npn-2 in *Npn-2* KO mice.

The light/dark transition, open field, and elevated plus maze tests have been widely used to assess anxiety-related behavior [15, 17, 18]. In the light/dark transition test, *Sema3F* KO mice exhibited increased latency to enter the light chamber, decreased distance traveled in the light and dark chambers, reduced number of transitions between the chambers, and stayed decreased time in the light chamber. *Sema3F* KO mice showed decreased distance traveled and reduced time spent in the center area in the open field during the early testing period, and also displayed decreased locomotor activity in the elevated plus maze. These data suggest that *Sema3F* KO mice show an innate aversion to a centain type of novel situations or increased anxiety to novelty. In addition, in the social interaction test, which has also been used to assess anxiety [19, 20], *Sema3F* KO mice showed decreased distance traveled and reduced duration of active social contact with a novel environment with a stranger mouse compared with WT controls. However, in the elevated plus maze test, there were no significant differences in the percentages of open time or open arm entries between *Sema3F* KO mice and WT controls. The absence of genotype effects on the behavioral indices in the elevated plus maze may be presumably explained by so called “floor effect” [21–23]. Overall, the results of the different types of tests for assessing anxiety-related behavior except for the elevated plus maze test suggest that Sema3F deficiency may induce abnormal anxiety-related behavior in novel environments, although further studies are needed to confirm whether Sema3F plays a critical role in the regulation of anxiety-related behavior in various situations.

In contrast to the reduced behavioral responses to the novel environments, *Sema3F* KO mice showed decreased immobility behavior in the Porsolt forced swim test, suggesting an antidepressant-like effect. However, this effect was not supported by the result of the tail suspension test. Similar findings were reported in mice deficient in phosphodiesterase 4 B, an enzyme that catalyzes hydrolysis of cyclic AMP [24], which is a potential downstream signaling molecule of class 3 Semaphorin [2]. It is reported that treatment of a specific agonist of corticotropin-releasing factor receptor subtype 1, stimulating the release of cyclic AMP, induced decrease in immobility in the forced swim test and anxiogenic-like effects in mice [25]. Since *Sema3F* KO mice exhibited abnormal anxiety-related behavior in some of tests, the ‘antidepressant-like’ effect observed in the forced swim test might be resulted from anxiety-related struggling, escape behavior, or heightened agitation in stressful situation, resulting in the decreased immobility. However, given that the two types of test are used as a measurement of depression-related behavior, further studies are needed to clarify the role of Sema3F in the regulation of depression-related behavior.

*Sema3F* KO mice showed no deficits in spatial reference learning or the long-term retention memory assessed by the Barnes maze test. On the other hand, in the contextual and cued fear conditioning test, we found that *Sema3F* KO mice displayed increased freezing responses in re-exposure to the conditioning context, indicating enhanced contextual fear memory. Moreover, *Sema3F* KO mice exhibited increased freezing when they were exposed to the altered context. This suggests that Sema3F deficiency induces enhanced generalized fear, reflecting impairment in the ability to distinguish two contexts, which is thought to be associated with a process known as pattern separation [26–28]. Our results seem to be consistent with those of Shiflett et al., reporting that *Npn-2* KO mice exhibited behavioral impairments in the two discrimination tasks based on object novelty and object or context association [5]. Thus, our findings suggest that Sema3F deficiency induces increased fear-related behavior or strengthens consolidation and generalization of fear memories for aversive experiences.

Sema3F/Npn-2 signaling is thought to contribute to development of neuronal circuit formation in the brain regions including hippocampus, cortex, olfactory bulb, and amygdala [8–14]. Tang et al. reported that mice deficient for COUP-TFII transcription factor showed abnormality of amygdala patterning, presumably due to defects in neuronal migration caused by decreased Npn-2 expression [29]. Sahay et al. demonstrated that mice deficient for Npn-2 or Sema3F showed severely disorganized and defasciculated axons of amygdala efferents projecting to the forebrain including the hypothalamus and the bed nucleus of the stria terminalis (BNST) [11]. Lesion and pharmacological studies have suggested that BNST is involved in the modulation of innate fear responses [30, 31]. These findings suggest that these anatomical defects, especially the abnormal targeting of amygdala efferents to BNST, may be associated with abnormalities in anxiety- and fear-related behaviors in *Sema3F* KO mice found in the present study. During synaptogenesis, Sema3F and its receptor Npn-2 are strongly expressed in the hippocampal DG [8, 12, 32]. In particular, Sema3F is abundantly expressed in the hilus of the DG and in the CA3 to which mossy and infrapyramidal tract (IPT) of the DG granule cells project, and Npn-2 is enriched in the molecular layer of the DG [12, 32]. Consistent with these observations, the hippocampal IPT projects abnormally to CA3 in *Sema3F* KO and *Npn-2* KO mice [11, 13]. It is reported that *Sema3F* and *Npn-2* KO mice exhibit increased spine number and size in the DG granule cells and cortical layer V pyramidal neuron and also increase frequency of miniature excitatory postsynaptic current, which indicate that Sema3F/Npn-2 signaling is a negative regulator of spine development and synaptic structure [12]. The density and size of spines in the hippocampus and cortex have been found to change after behavioral paradigms [33–38]. Increased spine morphogenesis and abnormal development of neural circuit induced by loss of Sema3F/Npn-2 signaling in various brain regions may induce increases in fear memory and generalized fear.

Disruptions of synaptic formation and function are associated with development of neurological and psychiatric disorders [39–41]. Recent human genetic studies have discovered associations between Semaphorins-neuropilins/plexins and various disorders, such as autism [42, 43], bipolar disorder [44], and schizophrenia [45–47]. However, so far it remains to be explored how these mutations lead to neurodevelopmental and functional outcomes of the brain. *Sema3F* KO mice may be served as a model to investigate the neural basis underlying some behavioral abnormalities related to the neurodevelopmental disorders.

## Conclusions

The present study provides novel findings on the behavioral effects of Sema3F deficiency through comprehensive behavioral analysis of *Sema3F* KO mice. We demonstrated that *Sema3F* KO mice show increased anxiety- and fear-related behaviors and enhanced fear memory, suggesting an important role of Sema3F in regulating innate and learned fear responses and memory functions. Sema3F/Npn-2 signaling is known to be crucial for hippocampal and cortical synaptic formation and organization during postnatal development. Thus, Sema3F can serve as a potential target to understand the neural mechanisms underlying neurodevelopment disorders and to treat the behavioral abnormalities.

## Methods

### Animals and experimental design

*Sema3F* KO mice were generated as previously described [8]. In brief, Sema3F loxP/+ mice were obtained by crossing *Sema3F* loxP-neo/+ mice with EIIa-cre transgenic mice [48]. Male *Sema3F* KO mice and the wild-type (WT) control littermates were generated from a cross between the heterozygous *Sema3F* loxP/+ mice with a C57BL/6 J background. Subjects were group-housed (four per cage, two KO and two WT) in a room with a 12-hr light/dark cycle (lights on at 07:00 hr) with access to food and water ad libitum. Behavioral testing was performed as previously described [49, 50]. The animals were 10–11 weeks old at the start of the testing. The tests were conducted between 09:00 and 17:00 hr. After the tests, all of the apparatus were cleaned with 70 % ethanol and super hypochlorous water to prevent a bias based on olfactory cues. All animal experiments were performed in accordance with the Guidelines for Animal Experimentation at Kobe University Graduate School of Medicine and Kyoto University Graduate School of Medicine. Behavioral tests were performed according to the test order as described below.

### General health and neurological screen

Physical characteristics, including body weight, rectal temperature, presence of whiskers or bald hair patches, were recorded. The righting, whisker twitch, and ear twitch reflexes were also evaluated. The neuromuscular strength was examined by the grip strength and wire hang tests as described previously [51]. The grip strength meter (O’Hara & Co., Tokyo) was used to assess forelimb grip strength. Mice were lifted and held by their tail so that their forepaws could grasp a wire grid, and then they were gently pulled backward until they release the grid. The peak force applied by mouse forelimbs was recorded in Newton. In the wire hang test, mice were placed on a wire mesh (O’Hara & Co., Tokyo), which was then inverted and waved gently, so that the subject grasped the wire. Latency to fall from the wire was recorded with a 60 sec cut-off time.

### Light/dark transition test

The light/dark transition test, developed by Crawley and colleagues [52], was performed as described previously [53]. The apparatus consisted of a cage (21 × 42 × 25 cm) divided into two sections of equal size by a partition with door (O’Hara & Co., Tokyo). One chamber was brightly illuminated (390 lux), whereas the other chamber was dark (2 lux). Mice were placed into the dark chamber, and allowed to move freely between the two chambers for 10 min after the door was open. The distance traveled (cm), total number of transitions, latency to first enter the light chamber (sec), and time spent in the light chamber (sec) were recorded automatically using ImageLD software (see ‘Image analysis’).

### Open field test

In the open field test [15], each subject was placed in the center of an open field apparatus (40 × 40 × 30 cm; Accuscan Instruments, Columbus, OH). Total distance traveled (cm), vertical activity (rearing measured by counting the number of photobeam interruptions), time spent in the center area (sec), and stereotypic counts (beam-break counts for stereotyped behaviors) were recorded. The center area was defined as 20 cm × 20 cm area located at the center of the field. Data were collected over a 120-min period.

### Elevated plus maze test

The elevated plus maze test, which has been widely used for evaluation of anxiety-related behavior [54, 55], was performed as described previously [56]. The apparatus consisted of two open arms (25 × 5 cm) and two enclosed arms of the same size with 15 cm high transparent walls, which arms were connected by a central square (5 × 5 cm) (O’Hara & Co., Tokyo). The arms and central square were made of white plastic plates and were elevated to a height of 55 cm above the floor. The open arms were surrounded by a raised ledge (3-mm thick and 3-mm high) to avoid mice falling off the arms. Arms of the same type were located opposite from each other. Each mouse was placed in the central square of the maze, facing one of the closed arms. The number of arm entries, distance traveled (cm), percentage of entries into open arms, and percentage of time spent in open arms were measured during a 10-min test period. Data acquisition and analysis were performed automatically using ImageEP software.

### Hot plate test

The hot plate test was performed to evaluate sensitivity to a painful stimulus. Mice were placed on a 55.0 ± 0.3 °C hot plate (Columbus Instruments, Columbus, OH), and latency to first hind-paw response was recorded. The hind-paw response was defined as either a paw lick or a foot shake.

### Social interaction test in a novel environment

Social interaction test was conducted to measure social behavior in a novel environment [20, 57]. Weight-matched (within 5 g) mice of the same genotype, which have been housed in different cages, were placed into an acrylic box together (40 × 40 × 30 cm) and allowed to explore freely for 10 min. The total number of contacts, total duration of contacts, total duration of active contacts, mean duration per contact, and total distance traveled were recorded and analyzed automatically using ImageSI software (see ‘Image analysis’). The active contact was defined as follows. Images were captured at 3 frames per second, and distance traveled between two successive frames was calculated for each mouse. If the two mice contacted each other and the distance traveled by either mouse was longer than 5 cm, the behavior was considered as an ‘active contact’.

### Rotarod test

Motor coordination and balance were tested with the rotarod test. Mice were placed on a rotating drum (3 cm diameter, UGO Basile Accelerating Rotarod). Time during which each animal was able to maintain its balance on the rod was measured (a cut-off time of 300 sec). The speed of the rotarod accelerated from 4 to 40 rpm over a 300-sec period.

### Startle response/prepulse inhibition tests

A startle reflex measurement system was used (O’Hara & Co., Tokyo). A test session began by placing a mouse in a Plexiglas cylinder where it was left undisturbed for 10 min. White noise (40 ms) was used as a startle stimulus. The intensity of startle stimulus was 110 or 120 dB. A prepulse sound was presented 100 msec before the startle stimulus, and its intensity was 74 or 78 dB. A test session consisted of six trial types (i.e. two types for startle stimulus only trials, and four types for prepulse inhibition trials). Four combination of prepulse and startle stimuli were employed (74-110, 78-110, 74-120, and 78-120 dB). Six blocks of the six trial types were presented in pseudorandom order such that each trial type was presented once within a block. The average interval was 15 sec (range: 10-20 sec). A 70 dB white noise was presented as a background noise during the test. Startle responses to the stimuli were recorded for 140 msec (measuring the response every 1 msec) starting with the onset of the prepulse stimulus. The peak startle amplitude recorded during the 140 msec sampling window was used as the dependent variable.

### Porsolt forced swim test

The Porsolt forced swim test, developed by Porsolt and colleagues [58, 59], was performed to assess depression-related behavior. Mice were placed into a Plexiglas cylinder (20 cm height × 10 cm diameter, O’Hara & Co., Tokyo) filled with water (23 °C), up to a height of 7.5 cm. Percentage of immobility and distance traveled (cm) were recorded over a 10-min test period. Data acquisition and analysis were performed automatically using ImagePS software (see Image Analysis).

### Contextual and cued fear conditioning test

To assess fear memory [60], mice were placed in a conditioning chamber (26 × 34 × 29 cm) in a sound-attenuated room and allowed to explore freely for 2 min. The animals were presented with an auditory cue (60 dB white noise) served as a conditioned stimulus (CS) for 30 sec. During the last 2 sec of the CS, mice were given a mild foot shock (2 sec, 0.5 mA) as an unconditioned stimulus (US). Two more CS-US pairings were presented with 120 sec inter-stimulus interval. Approximately 24 hr after the conditioning session, context test was performed in the conditioning chamber. Cued test in an altered context was performed after the context test using a triangular box (35 × 35 × 40 cm) made of white opaque plexiglas, which was located in a different sound-attenuated room. Following initial 3-min of pre-CS period, the CS was presented for 3 min. Data acquisition, control of stimuli (white noise and footshock), and data analysis were performed automatically using ImageFZ software. Images were captured at 1 frame per second. For each successive frame, the amount of area (pixels) by which the mouse moved was measured. When this area was below a certain threshold (i.e., 20 pixels), the behavior was judged as ‘freezing.’ When the amount of area equaled or exceeded the threshold, the behavior was judged as ‘non-freezing.’ The optimal threshold (the amount of pixels) to judge freezing was determined by adjusting it to the amount of freezing measured by human observation. ‘Freezing’ that lasted less than the defined time threshold (i.e. 2 sec) was not included in the analysis.

### Tail suspension test

Tail suspension test was used to evaluate depression-related behavior [61]. Mice were suspended 30 cm above the floor in a visually isolated area by adhesive tape placed approximately 1 cm from the tip of the tail. Immobility behavior was recorded for a 10-min test session. Data acquisition and analysis were performed automatically using ImageTS software.

### Barnes maze test

The Barnes circular maze task was conducted to test spatial reference memory [62] on ‘dry land’, a white circular surface, 1.0 m in diameter, with 12 holes equally spaced around the perimeter (O’Hara & Co., Tokyo). The circular open field was elevated 75 cm from the floor. A black Plexiglas escape box (17 × 13 × 7 cm), which had paper cage bedding on its bottom, was located under one of the holes. The hole above the escape box represented the target, analogous to a hidden platform in the Morris water maze task. The location of the target was consistent for a given mouse but randomized across mice. The maze was rotated daily, with the spatial location of the target unchanged with respect to the distal visual cues, to prevent a bias based on an olfactory cue or proximal cues within the maze. Three trials per day were conducted for 9 successive days. In each trial, latency to first reach the target hole (sec) and number of errors to reach the target hole was recorded by ImageBM software. On day 1 and 10, probe trials were performed without the escape box, to confirm that this spatial task was acquired based on navigation by distal environmental room cues. In the probe tests, time spent around each hole (sec) was measured using ImageBM software.

### Image analysis

All application softwares used for each behavioral analysis were run on Macintosh computers. The application softwares were based on the public domain NIH Image or Image J program (developed by Wayne Rasband at the National Institute of Mental Health, Bethesda) and were modified for each test by Tsuyoshi Miyakawa (available through O’Hara & Co., Tokyo).

### Statistical analysis

Statistical analysis was conducted using StatView (SAS Institute, Cary, NC). Data were analyzed by two-tailed t-tests or two-way repeated-measures ANOVAs. Values in graphs were expressed as mean ± SEM.
